# Retraction: “Evaluating the Clinical Efficacy of an Exergame-Based Training Program for Enhancing Physical and Cognitive Functions in Older Adults With Mild Cognitive Impairment and Dementia Residing in Rural Long-Term Care Facilities: Randomized Controlled Trial”

**DOI:** 10.2196/77638

**Published:** 2025-05-23

**Authors:** JMIR Editorial Office

**Affiliations:** 1 JMIR Publications, 130 Queens Quay E, Suite 1100, Toronto, ON, M5A 0P6, Canada, 1 416 583 2040

The JMIR Editorial Office is retracting the article “Evaluating the Clinical Efficacy of an Exergame-Based Training Program for Enhancing Physical and Cognitive Functions in Older Adults With Mild Cognitive Impairment and Dementia Residing in Rural Long-Term Care Facilities: Randomized Controlled Trial” by Li et al [1] following an investigation due to concerns over irregularities during the peer review process. We regret that these issues were not resolved prior to publication.

All authors disagreed with the retraction decision.
